# Supplementary material for: Evidence supported by Mendelian randomization: impact on inflammatory factors in knee osteoarthritis
Source: Front Med (Lausanne). 2024 May 28;11:1382836. doi: 10.3389/fmed.2024.1382836 (PMC11165061; doi:10.3389/fmed.2024.1382836)
Supplement: Supplementary file 1 [file Data_Sheet_1.ZIP › data/GCST90274820_5e6/820.leaveoneout.pdf]

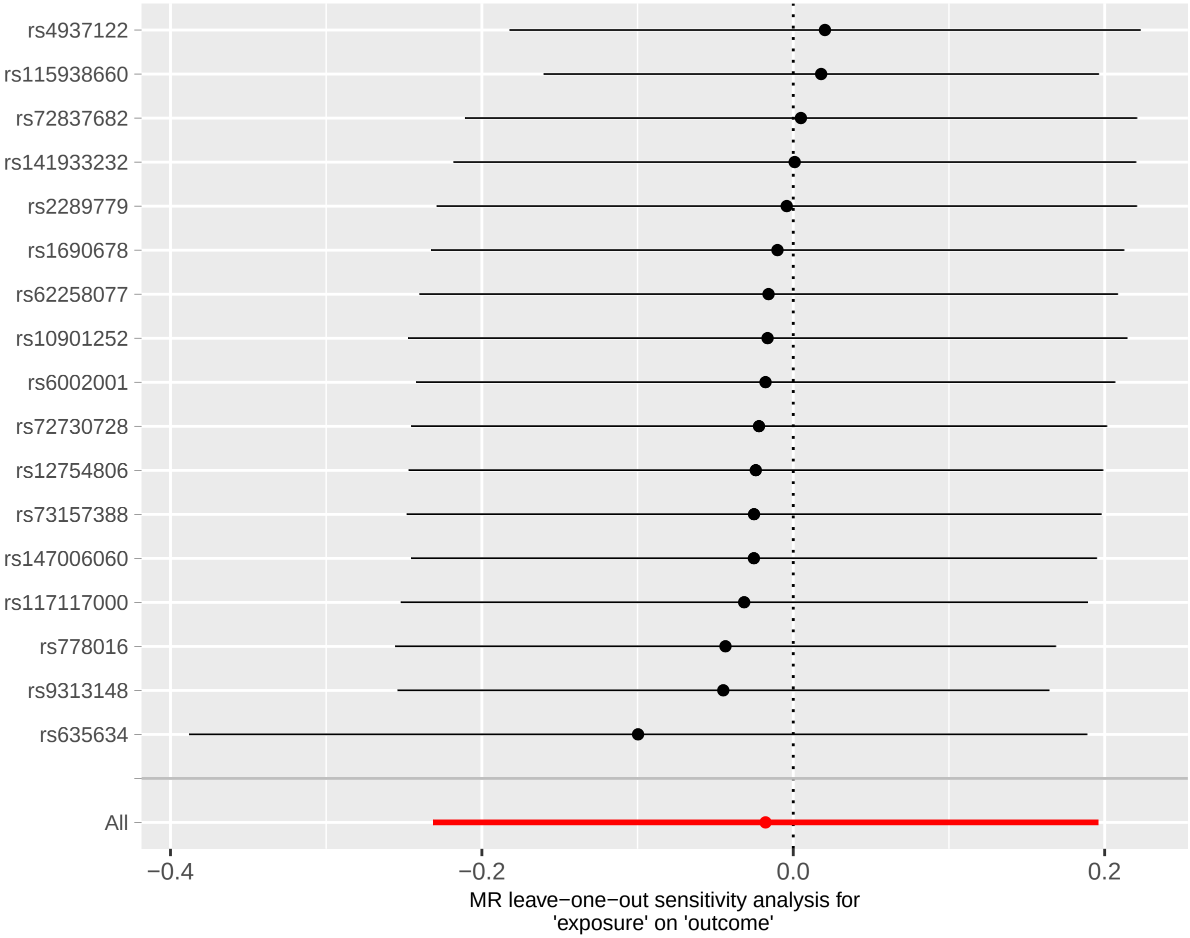

rs4937122
rs115938660
rs72837682
rs141933232
rs2289779
rs1690678
rs62258077
rs10901252
rs6002001
rs72730728
rs12754806
rs73157388
rs147006060
rs117117000
rs778016
rs9313148
rs635634
All
−0.4
−0.2
0.0
0.2
MR leave−one−out sensitivity analysis for
'exposure' on 'outcome'
